# Emergence of New ST301 Shiga Toxin-Producing *Escherichia coli* Clones Harboring Extra-Intestinal Virulence Traits in Europe

**DOI:** 10.3390/toxins13100686

**Published:** 2021-09-26

**Authors:** Aurélie Cointe, Etienne Bizot, Sabine Delannoy, Patrick Fach, Philippe Bidet, André Birgy, François-Xavier Weill, Sophie Lefèvre, Patricia Mariani-Kurkdjian, Stéphane Bonacorsi

**Affiliations:** 1 Service de Microbiologie, Centre National de Référence *Escherichia coli*, AP-HP, Hôpital Robert-Debré, Université de Paris, IAME, UMR 1137, INSERM, 75018 Paris, France; etienne.bizot@aphp.fr (E.B.); philippe.bidet@aphp.fr (P.B.); andre.birgy@aphp.fr (A.B.); patricia.mariani@aphp.fr (P.M.-K.); stephane.bonacorsi@aphp.fr (S.B.); 2 Platform Identy Path, Food Safety Laboratory, ANSES, Université Paris-Est, 94701 Maisons-Alfort, France; sabine.delannoy@anses.fr (S.D.); patrick.fach@anses.fr (P.F.); 3 Centre National de Référence des *Escherichia coli*, Shigella et Salmonella, Institut Pasteur, Unités des Bactéries Pathogènes Entériques, 75015 Paris, France; francois-xavier.weill@pasteur.fr (F.-X.W.); sophie.lefevre@pasteur.fr (S.L.)

**Keywords:** Shiga toxin, enterohemorrhagic *Escherichia coli*, heteropathotypes, ST301, pS88 plasmid, hemolytic and uremic syndrome, extra-intestinal virulence, genetic plasticity

## Abstract

O80:H2 enterohemorrhagic *Escherichia coli* (EHEC) of sequence type ST301 is one of the main serotypes causing European hemolytic and uremic syndrome, but also invasive infections, due to extra-intestinal virulence factors (VFs). Here, we determined whether other such heteropathotypes exist among ST301. EnteroBase was screened for ST301 strains that were included in a general SNP-phylogeny. French strains belonging to a new heteropathotype clone were sequenced. ST, hierarchical clusters (HC), serotype, resistome, and virulome were determined using EnteroBase, the CGE website, and local BLAST. The ST301 general phylogeny shows two groups. Group A (*n* = 25) is mainly composed of enteropathogenic *E. coli*, whereas group B (*n* = 55) includes mostly EHEC. Three serotypes, O186:H2, O45:H2 and O55:H9, share the same virulome as one of the O80:H2 sub-clones from which they derive subsequent O-antigen switches. The O55:H9 clone, mainly present in France (*n* = 29), as well as in the UK (*n* = 5) and Germany (*n* = 1), has a low background of genetic diversity (four HC20), although it has three Stx subtypes, an H-antigen switch, and genes encoding the major extra-intestinal VF yersiniabactin, and extended-spectrum beta-lactamases. Diverse heteropathotype clones genetically close to the O80:H2 clone are present among the ST301, requiring close European monitoring, especially the virulent O55:H9 clone.

## 1. Introduction

Enterohemorrhagic *Escherichia coli* (EHEC) infections represent a major public health concern because of their outbreak potential and the severe morbidity related to hemolytic uremic syndrome (HUS). This complication, associated with 3% to 5% mortality worldwide [1], results from the action of the Shiga toxin (Stx) on its target organs, in particular the intestine, kidneys, and brain. Another major virulence factor (VF) in typical EHEC is the intimin protein (*eae*), which is involved in intestinal adhesion with the effacement of microvilli, leading to so-called A/E lesions. EHEC are also characterized by the presence of the plasmid-borne enterohemolysin gene (*ehxA*).

Hybrid pathotype clones, also called heteropathotypes, have recently emerged in Europe, such as the Enterohemorrhagic–Uropathogenic *E. coli* (EHEC-UPEC) strain, first described among the O2:H6 serotype [2], or the Enterohemorrhagic–Extraintestinal pathogenic *E. coli* (EHEC-ExPEC) strain of serotype O80:H2. Initially described in France [3,4], serotype O80:H2 emerged in the 2010s to become one of the three major serotypes involved in HUS cases [5]. It has recently also been reported to be present in several countries [6,7,8,9] and now represents the third most frequent serotype isolated from HUS cases in Europe, responsible for 9% of European HUS in 2019 [10].

This emerging clone, belonging to sequence type (ST) 301 and clonal complex (CC) 165, forms a separate group in the EHEC phylogeny, as it belongs to phylogroup A [11], whereas the others belong to phylogroup E (O157:H7 and O55:H7) or B1 (non O157/O55:H7 serotypes) [12], suggesting that the O80 clone emerged independently from the other serogroups. All reported O80:H2 EHEC strains carry a rare subtype of intimin, *eae-ξ*, first described in a Stx producing-*E. coli* (STEC) isolated from Spanish cattle [13] and shared with Enteropathogenic *E. coli* (EPEC) O80:H2 isolated from humans [11] or young diarrheic calves [7,14,15], and can thus be considered a O80:H2 clone hallmark. The heteropathotype O80 clone combines classical intestinal VFs (presence of the *stx*, *eae-ξ*, and *ehxA* genes) and plasmid-borne extra-intestinal virulence determinants. This plasmid (pR444_A) shows strong homology with the pS88 plasmid, known to be involved in neonatal meningitis and to be present in Avian Pathogenic *E. coli* (APEC). Compared to pS88, an additional antibiotic resistance cassette that confers resistance to penicillins, cotrimoxazole, tetracyclines, streptomycin, kanamycin, and heavy metals, such as mercury, have been integrated [11]. In addition to digestive damage and HUS, the O80 clone can cause invasive infections, such as bacteremia or deep abscesses [3,4], which may be due to the presence of this mosaic plasmid (pR444_A). Most O80:H2 EHEC strains also carry a cryptic plasmid (pR444_B) of unknown function belonging to the phage-like plasmid family [11].

We recently identified an EHEC strain of serotype O55:H9 (45057) harboring *eae-ξ* and belonging to ST301 [16]. This strain appears to be genetically very close to the O80:H2 clone, as it shares the same extra-intestinal VFs and antibiotic resistance determinants, suggesting that the pS88-like plasmid can be present in isolates other than O80:H2.

Here, we aimed to determine whether this new O55:H9 heteropathotype strain is unique or if other heteropathotype clones exist within ST301, a clonal group that includes all the characteristics that allow the extensive dissemination of strains with high disease potential. We evaluated the extent of the diffusion of the pS88-like plasmid in this particular ST301 by screening EnteroBase. We then focused on the O55:H9 subgroup, which appears to have emerged in France and possibly other countries, and studied its relationship with the O80:H2 clone. Improving our knowledge of heteropathotype strains is essential, given their potential invasiveness, which makes them a therapeutic challenge and a threat that requires close monitoring.

## 2. Results

### 2.1. ST301 Characterization and Phylogeny

**Figure 1:**
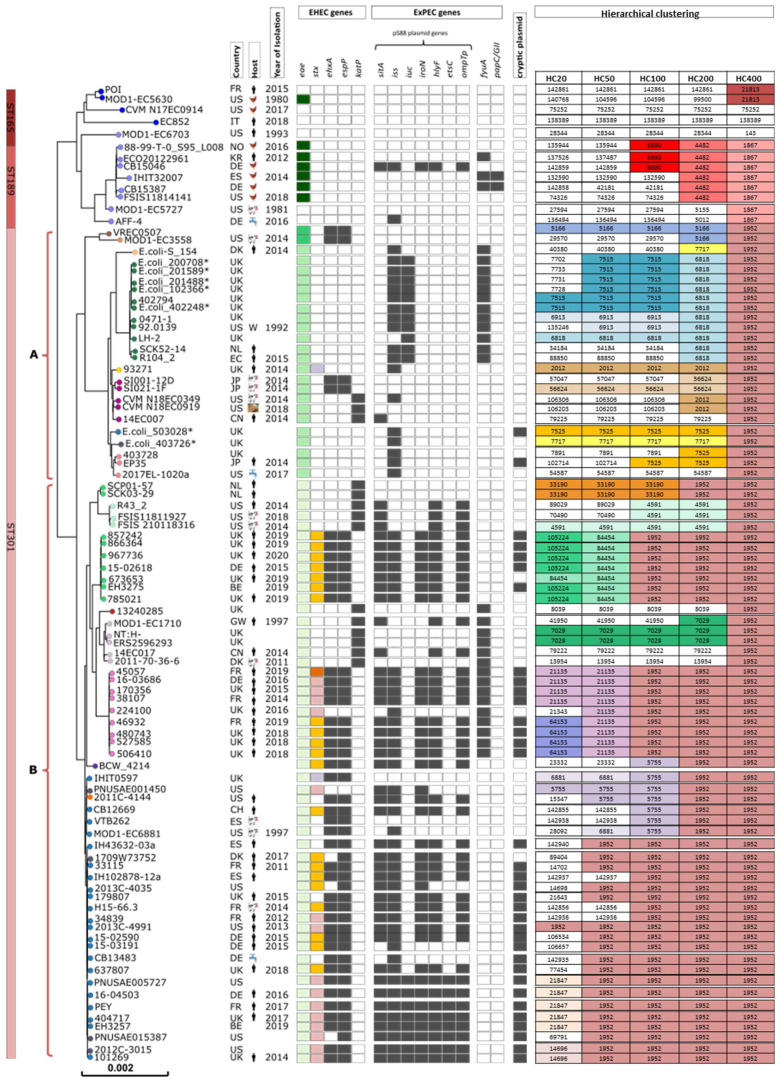

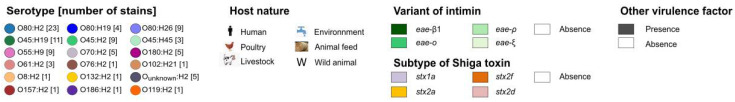
SNP-Phylogeny and hierarchical clustering of 93 strains belonging to the clonal complex CC165, isolated from various sources and countries throughout the world between 1980 and 2020. Caption: A maximum-likelihood tree based on 37,956 core SNPs within all 93 genomes retrieved from EnteroBase was built. Clonal complex (CC) 165 is divided into three STs (ST165, ST189, and ST301), as indicated by the colored rectangle on the left side of the figure. ST301 is split into two groups, A and B. The origin of the strain (country, year, and source of isolation) is represented, if available, by the suitable abbreviation (BE, Belgium; CN, China; DE, Germany; DK, Denmark; EC, Ecuador; ES, Spain; FR, France; GW, Guinea-Bissau; IT, Italy; JP, Japan; KR, Korea; NL, Netherlands; NO, Norway; UK, United Kingdom; US, USA) and symbols for human, animal (picture, W for wild animal or haystack for a feed animal), and water for environmental source. The strain serotype is represented by a colored circle to the left of the name of the strain. The presence of intimin (*eae*) and Shiga toxin (*stx*) genes are represented by a colored square, depending on the nature of the subtype. The presence of virulence genes or the cryptic plasmid (pR444_B) is indicated by a black square. The genomes were also assigned to hierarchical clusters (HCs) based on core genome MLST to assess their genomic relatedness. Common HCs are represented by a number highlighted in the same color, indicating that the strains are closely related. For example, two strains sharing the same HC10 means they have links that are no more than 10 alleles apart. * signifies that the name of the strain has been shortened for the figure (*n* = 7). The complete names are as follows: *Escherichia_coli*_200708-sc-2012-01-13T20:11:28Z-1332516, *Escherichia_coli*_201589-sc-2012-01-13T20:11:52Z-1332550, *Escherichia_coli*_201488-sc-2012-01-13T20:11:51Z-1332549, *Escherichia_coli*_102366-sc-2012-01-13T20:11:47Z-1332544, *Escherichia_coli*_402248-sc-2012-01-13T20:11:08Z-1332489, *Escherichia_coli*_503028-sc-2012-01-13T20:11:16Z-1332499, and *Escherichia_coli*_403726-sc-2012-01-13T20:11:40Z-1332534.

We determined whether other heteropathotypes are present within ST301 (CC165) by screening the EnteroBase database and identified 301 ST301 strains (accessed on 5 August 2020). Most (72%) belonged to O80:H2 (*n* = 218/301), of which two thirds were isolated in France (*n* = 145). We previously showed that these French strains form a highly homogeneous clonal group, regardless of their origin [11]. Thus, only a small representative sample (*n* = 23), mainly from foreign countries, was included for the phylogenetic study. On the contrary, all sequences of non-O80:H2 ST301 strains were included (*n* = 57/301), with the exception of the French O55:H9 EHEC for which 3/29 representative strains were included. We also added 13 CC165 O80 strains belonging to ST165 (*n* = 5/14) and ST189 (*n* = 8/30) to be used as outgroups [11]. A maximum-likelihood tree was inferred from the 37,956 core SNPs present in the 93 (80 + 13) genomes analyzed (Figure 1). The genomes were also assigned to HCs based on core genome MLST using EnteroBase to assess their genomic relatedness.

The 93 strains were isolated between 1980 and 2020 from various regions of the world, with 15 submitting countries. Among the available data (69/93), half of the strains came from humans (46/93), followed by livestock (11/93) and poultry (7/93). Although the serogroups varied, we observed a large predominance of flagellar antigen H2 (12 of the 18 serotypes found).

All O80 ST165 strains (*n* = 5) had no VF, except for one (MOD1-EC5630) carrying the intimin gene (*eae-β1*) alone. The ST189 group contained six O80 EPEC strains, characterized by the β1 subtype of intimin (*eae-β1*). A German EPEC (CB15046) acquired extra-intestinal virulence genes characteristic of the pS88-plasmid (EPEC-ExPEC) and two other strains (IHIT32007 and CB15387) harbored uropathogenic *papC/GII* genes (EPEC-UPEC). However, no strain of ST165 or ST189 carried Shiga toxin genes (*stx*) (Figure 1).

All ST301 (*n* = 80) strains shared the same HC400, that is, HC400_1952, meaning that they have links that are no more than 400 alleles apart. Our phylogenetic analysis revealed two groups, named A and B, within this ST (Figure 1). Group A (*n* = 25) strains mainly carried the rho subtype of *eae* (*eae-ρ*), whereas all group B strains (*n* = 55) shared the same subtype, *eae-ξ*, characteristic of the O80:H2 clone, regardless of their serotype. Moreover, no strain of group A, except one (93271), harbored Stx encoding genes, whereas 69% of group B strains (38/55) were EHEC, characterized by the presence of four different *stx* subtypes (*stx1a*, *stx2a*, *stx2d*, or *stx2f*) associated with the enterohemolysin encoding-gene (*ehxA*) in 33 strains.

Group A is formed by strains from a variety of sources belonging to nine distinct serotypes (mainly O45:H19, O180:H2, and O61:H2), with HC400 as the smallest common HC (Figure 1). Group B is formed mainly by strains isolated from humans (36/55) and contains, aside from O80:H2, eight different serotypes (O55:H9, O45:H2, O70:H2, O45:H45, O_unknown_:H2, O157:H2, O186:H2, and O119:H2). It appears to be less heterogeneous than group A, as two HC100 clusters (HC100_1952 and HC100_5755) are shared between four of the eight distinct serotypes (O80:H2, O186:H2, O55:H9, and O45:H2). Of interest, these four serotypes include strains with heteropathotype characteristics.

Within the O45:H2 subgroup (*n* = 9), seven *stx2a* carrying *E. coli* strains appear to be heteropathotypic, as they also carried five of the seven VFs characteristic of the pS88-like plasmid (incomplete pS88-like plasmid). An identical virulence profile was present in a O186:H2 strain (BCW_4214). The O55:H9 subgroup consisted of nine human EHECs carrying *stx2f* (*n* = 1), *stx2d* (*n* = 4), or *stx2a* (*n* = 4). This subgroup, except for one strain (224100 has no pS88 VFs), also contained the incomplete pS88-like plasmid. Interestingly, this subgroup harbored yersiniabactin-encoding genes, represented by *fyuA*. The O80:H2 subgroup is by far the main representative of the ST301 clonal group and we selected a set of 23 representative strains for this study. In our panel, the O80:H2 strains possessed either *stx2a* (*n* = 8) or *stx2d* (*n* = 8), except for one strain (IHIT0597) harboring *stx1a*. There were two types of pS88-like plasmids in this serotype, the incomplete and the complete which appears to be restricted to certain O80:H2 *stx2d*-producing strains. No O80:H2 strain possessed yersiniabactin.

Overall, this phylogenetic analysis shows that heteropathotype *E. coli* combining intestinal and extra-intestinal virulence are not solely represented by the O80:H2 clone. Three serotypes (O186:H2, O55:H9, O45:H2) shared the same virulome as the O80 clone (*stx*, *eae-ξ*, *ehxA*, and incomplete pS88-like plasmid), associated with the cryptic plasmid (pR444_B) for two of them. These new heteropathotypes have spread to at least four European countries. The close phylogenetic relationship between these strains and the O80:H2 clone is shown by a common HC100 cluster (HC100_1952 or HC100_5755), whereas all other ST301 strains share the same HC400 (Figure 1). Although we showed the existence of three other heteropathotype serotypes in Europe, only the O55:H9 strains were isolated from humans in France between 2014 and 2019. O55:H9 currently represents the second most frequently isolated heteropathotype clone after O80:H2. We thus focused on this serotype and its relationship with the O80:H2 clone.

### 2.2. The O55:H9 Serotype and Its Relationship with the O80 Serogroup

**Figure 2:**
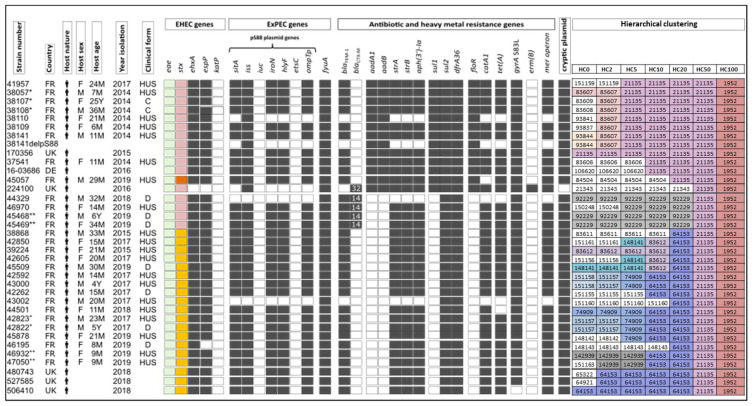
SNP-Phylogeny and hierarchical clustering of 35 clinical O55:H9 EHEC isolated from Humans in Europe between 2014 and 2019. Caption: The origin of the strain (country, year, and source of isolation) is represented, if available, by the suitable abbreviation (DE, Germany; FR, France; UK, United Kingdom) and the symbol of a human. The clinical form is indicated by a code as follows: HUS, hemolytic uremic syndrome; C, carrier; and D, (bloody)-diarrhea. Demographic and clinical data are missing for the non-French strains. Among these strains, seven are linked, as they were isolated from three families (38057*, 38107*, and 38108*; 45468** and 45469**; and 45822+ and 45823+). Two strains were isolated from the urine and stool from the same patient without signs of urosepsis (46932++ and 47050++, respectively). The presence of intimin (*eae*) and Shiga toxin (*stx*) genes is represented by a colored square, depending on their subtype (*eae-ξ*, green; *stx2a*, yellow; *stx2d*, pink; and *stx2f*, orange). The presence of virulence and antibiotic-resistance genes or the cryptic plasmid (pR444_B) is indicated by a black square. The numbers in the squares indicate the variant of *bla*_CTX-M_. The genomes were also assigned to hierarchical clusters (HCs) based on core genome MLST to assess their genomic relatedness. Common HCs are represented by a number highlighted in the same color, indicating that the strains are closely related. For example, two strains sharing the same HC10 means they have links that are no more than 10 alleles apart. 38141delpS88 is the 38,141 mutant cured of its pS88-like plasmid using SDS.

We fully characterized all French O55:H9 EHEC strains isolated by the FNRC (*n* = 29) and compared them to the six other European strains present in EnteroBase (Figure 2). This serotype was identified in France in 2014 and in other European countries in 2015. The at-risk population of this serotype is identical to that of other serotypes involved in HUS in France [17], with slightly more female patients (52%, *n* = 14/27) and a mean age of 22.8 months (excluding the two strains isolated from asymptomatic carriers). More than two-thirds of the cases were HUS (*n* = 20/29), a rate comparable to that of O80:H2 for the same period (66.3%, *n* = 128/193, FNRC data). Unlike O80:H2, no invasive infections were observed for O55:H9 EHEC.

All O55:H9 strains were typical EHEC, combining the intimin gene *eae-ξ*, a subtype of *stx* (mainly *stx2d* or *stx2a*), and the plasmid-borne enterohemolysin gene (*ehxA*), with the exception of one strain (224100). Genetic markers of the incomplete pS88-like plasmid were present in 89% of O55:H9 EHEC (31/35). Moreover, 91% (32/35) of O55:H9 EHEC possessed at least four of five antibiotic-resistance genes (MDR) conferring resistance to penicillins (*bla*_TEM-1_), streptomycin (*strA*, *strB*), kanamycin (*aph(3′)-Ia*), cotrimoxazole (*sul*, *dfrA*), or tetracycline (*tet*), as well as heavy-metal resistance genes (*mer* operon). All strains, except one (506410), harbored a mutation in the quinolone-resistance determining region of the DNA gyrase (*gyrA S83L*), conferring quinolone resistance, as described for O80:H2 strains [11]. Of note, five O55:H9 EHEC (224100, 44329, 46970, 45468, and 45469) contained an additional extended-spectrum beta-lactamase (ESBL) gene (*bla*_CTX-M-32_, *bla*_CTX-M-14_). In terms of antibiotic resistance, two main profiles could be defined by the presence or absence of six additional resistance genes (*aadA1*, *aadB*, *sul1*, *sul2*, *dfrA36*, and *floR*). The presence of these genes in strain 38110, which lacks the pS88-like plasmid, suggests that they are located on the bacterial chromosome (Figure 2). Sequence analysis of the pS88-like plasmid-cured strain (38141delpS88) allowed us to confirm that these six antibiotic-resistance genes are present in a chromosomal gene cassette.

The almost perfect concordance between the presence/absence of plasmid-borne VFs and the other antibiotic-resistance factors (33/35 strains) suggests the presence of a mosaic plasmid in the O55 clone, similar to that previously described for the O80 clone (pR444_A) [11]. Indeed, co-localization of extra-intestinal VFs and MDR genes on the same plasmid was confirmed both by Southern hybridization for strain 38141 (data not shown) and the sequence of its isogenic strain cured of the pS88-like plasmid (38141delpS88) (Figure 2). We assume that cryptic plasmid (pR444_B), first described in the RDEx444 strain [11], is also carried by O55:H9 EHEC, as >85% of its genetic determinants are present in all but one (224100) strain. A strong difference between these two serogroups is the presence of the yersiniabactin *fyuA* gene, shared by all O55:H9 strains and never present in O80:H2.

These common features all highlight the extreme genetic similarity between the European O55:H9 and O80:H2 EHEC strains, suggesting the existence of a common ancestor for both serotypes, from which they emerged at different times and then evolved independently. Concerning the hierarchical clustering analysis, there is stronger genetic homology among the O55 group. All European O55 strains harbored the same HC50 (HC50_21135) and three lineages could be distinguished based on HC20, excluding the atypical strain 224100. All the strains of HC20_21135 (*n* = 11) harbored an Stx2d subtype except one (45057), which carried Stx2f. Almost two-thirds of these strains (*n* = 7/11) share strong genetic homology, shown by a common HC2_83607, whereas an epidemiological link could only be established for three of these seven strains. The four Stx2d-producing strains carrying an ESBL (*bla*_CTX-M-14_) form the second subgroup, characterized by an HC20_92229. The 19 Stx2a-producing strains constitute the third subgroup (HC20_64153) (Figure 2).

Overall, O55:H9 is a highly homogeneous group, as several strains, even without apparent familial transmission, share a common HC2 or even HC0, reflecting the occurrence of outbreaks (Figure 2).

## 3. Discussion

Here, we show that among ST301, three new serotypes of *E. coli* (O55:H9, O186:H2, O45:H2) harbor heteropathotype traits comparable to those of the historical O80:H2 clone and are, thus, perfectly equipped to induce both HUS and extra-intestinal infections. Among them, the O55:H9 clone has been emerging in France and is also present in several other European countries. It has rapidly evolved, acquiring various virulence and antibiotic-resistance traits.

The ST301 clonal group appears to be highly diverse based on the 16 observed serotypes, although the H2 flagellar antigen is present in 75% of cases (12/16). As previously shown [18], flagellar-antigen loci recombine less frequently than the somatic-O antigen gene cluster. Therefore, the H2 antigen may be the ancestral flagellin of ST301, whereas numerous switches of the O antigen gene cluster have occurred in this ST.

Our study allowed us to obtain a comprehensive view of ST301 and identify two major groups—one (group A) mainly consisting of atypical EPEC, devoid of the bundle-forming pili-encoding gene (*bfpA*), and the other (group B) mainly consisting of heteropathotype *E. coli*. Ninety-two percent (23/25) of the atypical EPEC of group A carried *eae-ρ*, first described in 2006 in a O21:[h5] EPEC strain [19]. The *eae* subtypes γ1, β1, ε, and θ are known to be associated with major EHEC serogroups [20], whereas the *eae-ρ* subtype is uncommon [21] and exceptionally associated with *stx* genes, especially in humans, with only one strain reported to date (O180:H2, *stx2a*, ST301) [22]. We thus report the second EHEC harboring *eae-ρ* (93271), associated here with the *stx1a* subtype. The reason ST301 strains carrying the *eae-ρ* subtype almost never harbor *stx* genes, in contrast to those carrying *eae-ξ*, is yet to be elucidated.

The second group (B), dominated by the O80:H2 cluster, is likely derived from a common ancestor, an atypical EPEC of serotype O_XX_:H2 carrying *eae-ξ*, which has evolved into several lineages. Among them, the O80:H2 clone and three other serotypes (O55:H9, O186:H2, and O45:H2) share a common HC100 and harbor *stx* and other virulence genes essential for EHEC pathogenesis (*ehxA*) and virulence traits characteristic of the pS88-like plasmid, as well as the cryptic plasmid (pR444_B), whereas none of these combined features are present in the other group B lineages. The common DNA gyrase mutation(*gyrAS83L*) and the presence of the mercury-resistance operon (*mer*)—both hallmarks of the O80:H2 clone [11]—are also shared by these serotypes (data not shown). Two forms of the pS88-like plasmid, that is, the complete version with seven VFs and the incomplete one with five, were found in these heteropathotype strains. However, the complete form exists only in the O80:H2 serotype. Long-read sequence analysis of an American O80:H2 EHEC carrying the incomplete pS88-like plasmid (2013C-4991) [23] allowed us to find the exact same chromosomal resistance stigma (*aadA1*, *aadB*, *sul1*, *sul2*, *dfrA36*, and *floR*) as those described in the O55:H9 strain (38141delpS88), which is a further argument for a common genetic background.

**Figure 3:**
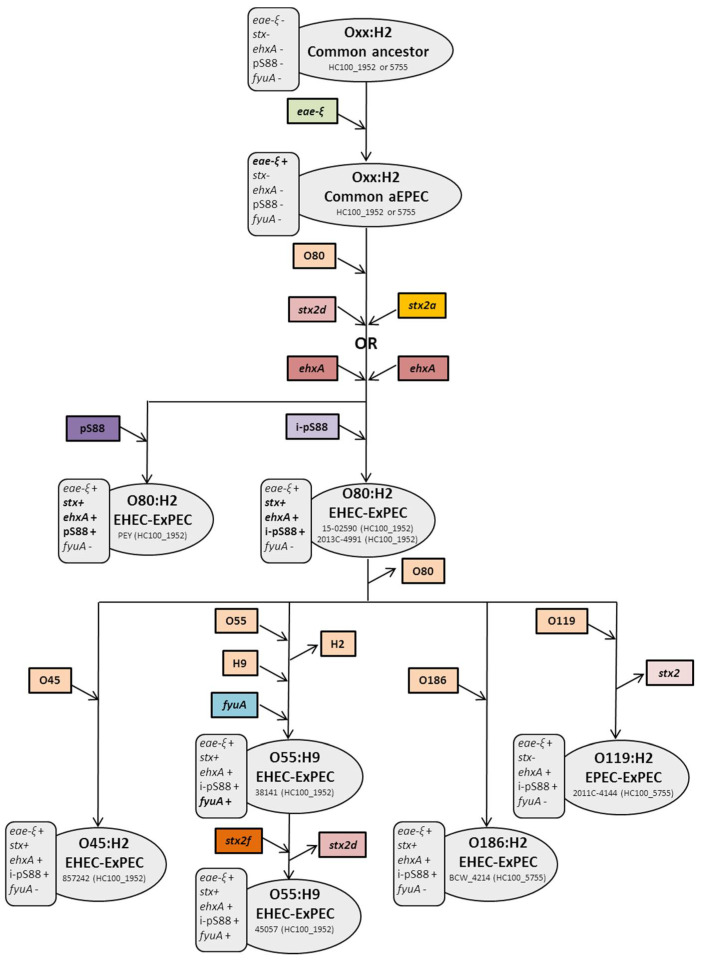
Hypothetical scenario for the evolution of ST301 from a common HC100 background to diversification into various serotype leading to new heteropathotype clones. Caption: An example of the strain is given for each step. aEPEC, atypical enteropathogenic *Escherichia coli*; EHEC, enterohemorrhagic *Escherichia coli*; ExPEC, extra-intestinal *Escherichia coli*; *eae*, intimin; *stx*, Shiga toxin; *ehxA*, enterohemolysin; pS88, complete form of the pS88-like plasmid; i-pS88, incomplete form of the pS88-like plasmid; *fyuA*, yersiniabactin; + indicates the presence of the gene, whereas—indicates its absence; an arrow pointing downwards indicates the acquisition of a VF, whereas an upward-pointing arrow illustrates its loss.

The most plausible hypothesis, suggested by all these common genetic features, is that the new heteropathotype strains are derived from the O80:H2 subgroup carrying the incomplete plasmid form. They diverged from their common HC100 background and subsequently diversified into various serotypes by changing their O antigen. Interestingly, an American strain of serotype O119:H2 (2011C-4144), carrying the *eae-ξ* and *ehxA* genes, embedded within the O80:H2 group and sharing its HC100_5755, provides another example of an O antigen switch. Of note, this strain could be another heteropathotype that subsequently lost the *stx*-encoding prophage. A hypothetical scenario describing the evolutionary history of ST301 is presented in Figure 3.

Of interest, a very recent study reported the presence of new heteropathotype strains in Italy and the Netherlands, confirming their current European diffusion [24]. Several O186:H2 (*n* = 3), isolated between 2000 and 2007, and O45:H2 (*n* = 4), isolated between 2017 and 2019, *stx2a*-producting EHECs were described in this Italian study but only one O55:H9 *stx2f* strain isolated in 2014 was reported [24] (not publicly available at time of writing), in contrast to the 29 French strains studied here. A maximum of ten allelic differences (HC10) were observed between English, German and French O55:H9 strains, which indicates a very close relationship. More broadly, all the European O55:H9, depicted in Figure 2, are closely related, sharing a common HC50, and several appear to belong to clusters even without apparent familial transmission. Indeed, the presence of a common HC2, or even HC0, reflects the occurrence of outbreaks. However, as for the O80:H2 strains, the source of contamination is yet to be clearly identified. The lower diversity observed of O55:H9 relative to that of O80:H2 suggests a more recent origin for O55:H9. At the European level, the current methodology for STEC detection in food is substantially harmonized as most laboratories rely on the international standard ISO TS 13136:2012. This method includes the detection of STEC VFs (*stx*, *eae*) and genes associated with the five historical major serogroups (O157, O26, O145, O111 and O103) [25]. However, it is now accepted that all STEC strains, regardless of their serotype, may be associated with human severe illness. The description of such heteropathotype clones, with potential invasiveness, reinforces the need for a broader screening of food matrices in industry.

Aside from its recent diffusion in several European countries, the heteropathotype O55:H9 cluster is remarkable for its high genetic plasticity, leading to an H antigen change, *stx*-subtype switching, and the acquisition for ESBL-encoding genes, as well as chromosomal integration of genes encoding yersiniabactin (*fyuA*). The acquisition of this iron-uptake system can provide this clone with an additional advantage for extraintestinal virulence, as it has been reported to surpass other siderophores [26] such as the aerobactin and the *sitABCD* operons, as shown by higher mortality in a mouse model of sepsis. Although no extraintestinal infections have occurred thus far, this heteropathotype clone is perfectly equipped to cause life-threatening invasive infections.

## 4. Conclusions

In conclusion, our study showed the pS88-like plasmid to be present within ST301, revealing the European spread of at least three new heteropathotype serotypes (O186:H2, O55:H9, and O45:H2) with the same virulome as the O80:H2 clone. SNP-phylogeny, hierarchical clustering and a common arsenal of mobile genetic elements suggest that these new heteropathotypes are derived from the O80:H2 cluster. The emergence and spread of these new heteropathotype clones are of particular concern, suggesting that they may follow the same epidemiological evolution as the O80:H2 clone. Among these new clones, O55:H9 dominates and appears to have recently emerged with the exceptional ability to acquire new genomic information carried by mobile genetic elements through horizontal gene transfer. This versatile O55:H9 serotype appears to be of particular interest and requires close monitoring.

## 5. Materials and Methods

### 5.1. Strains, Strain Sequences from a Database, and Sampling

EnteroBase (http://enterobase.warwick.ac.uk/species/index/ecoli) was screened to search for all ST301 (CC165) *E. coli* strains. The number of sequences available in EnteroBase (accessed on 5 August 2020) and that of those included in the study are summarized in Table S1. All French O55:H9 strains (*n* = 29) were isolated from the French National Reference Center (FNRC) of *E. coli*. A variant of strain 38141 (O55:H9) cured of its pS88-like plasmid (38141delpS88) was obtained as previously described [27]. Thirteen O80 strains belonging to CC165 but not ST301 were also included: five of ST165 (4/13 O80:H19, 1/1 O80:H26) and eight of ST189 (8/30 O80:H26). All sampling was conducted according to the phylogenetic representativeness, *stx* subtype and temporal, demographic and geographic origin of the strains. The information for each strain (temporal and geographical origin, nature, sex and age of the host, serotype, and clinical involvement) was collected from EnteroBase or the FNRC database and is depicted in Figure 1 and Figure 2.

### 5.2. Whole Genome Sequencing

All French O55:H9 strains (*n* = 29) and the strain cured of the pS88-like plasmid (38141delpS88) were sequenced for this study, except for one (45057) that was part of a previous study [16]. WGS was carried out at the Plateforme de Microbiologie Mutualisée (P2M) of the Pasteur International Bioresources network (PIBnet, Institut Pasteur, Paris, France). The MagNAPure 96 system (Roche Diagnostics, Indianapolis, IN, USA) was used for DNA extraction, the libraries prepared using the Nextera XT kit (Illumina, San Diego, CA, USA), and sequencing performed using the NextSeq 500 system (Illumina). Genomes were assembled within EnteroBase using the available standard pipelines [28]. The statistics of each sequenced genome are summarized in Supplementary Table S2. The nucleotide sequences were deposited in GenBank (Bioproject no. PRJN693242) and EnteroBase.

### 5.3. Sequence Analysis

Serotype Finder [29] was used if the serotype was missing in EnteroBase. Achtman MLST typing and investigation of the resistome were performed using the CGE website [30] Genomes were assigned to hierarchical clusters (HCs) on the basis of the number of pair-wise allelic differences in the core genes using EnteroBase [28]. Genes of intestinal and extraintestinal VFs were assessed by local BLAST+ 2.2.31 analysis [31], as previously described [11]. The presence of cryptic plasmid was defined by a blast leading to ≥99% nucleotide identity for ≥85% coverage with the pR444_B plasmid (NZ_QBDM01000003.1).

### 5.4. pS88-like Plasmid

This plasmid is characterized by the presence of genes encoding aerobactin [*iuc*], salmochelin [*iroN*], an iron-uptake protein [*sitABCD*], a serum-resistance protein [*iss_p_*], a putative secretion system I [*etsC*], an omptin T [*ompT_p_*], and a hemolysin [*hlyF*] strongly related to that of the pS88 plasmid [27], known to be involved in neonatal meningitis and to be present in APEC. The absence of the *iuc* and *etsC* genes results in an “incomplete” form of this plasmid.

### 5.5. Phylogeny

A maximum-likelihood tree based on single nucleotide polymorphism (SNP) differences of the 93 genomes (80 ST301 strains and 13 non-ST301 strains but belonging to CC165) was generated using EnteroBase [28].

## Supplementary Materials

The following are available online at https://www.mdpi.com/article/10.3390/toxins13100686/s1, Table S1: Number of ST301 sequences available in EnteroBase (accessed on 5 August 2020) and that of those included in this study, Table S2: Statistic of the 29 French O55:H9 EHEC genomes sequenced in this study.
