# Supplementary material for: Emergence of New ST301 Shiga Toxin-Producing Escherichia coli Clones Harboring Extra-Intestinal Virulence Traits in Europe
Source: Toxins (Basel). 2021 Sep 26;13(10):686. doi: 10.3390/toxins13100686 (PMC8537712; doi:10.3390/toxins13100686)
Supplement: Supplementary file 1 [file toxins-13-00686-s001.zip › toxins-1374232-supplementary.pdf]

toxins

MDPI

Article

Emergence of New ST301 Shiga Toxin-Producing *Escherichia-coli* Clones Harboring Extra-Intestinal Virulence Traits in Europe

Aurélie Cointe [1,*], Etienne Bizot [1], Sabine Delannoy [2], Patrick Fach [2], Philippe Bidet [1], André Birgy [1], François-Xavier Weill [3], Sophie Lefèvre [3], Patricia Mariani-Kurkdjian [1] and Stéphane Bonacorsi [1]

[1] Service de Microbiologie, Centre National de Référence *Escherichia coli*, AP-HP, Hôpital Robert-Debré, Université de Paris, IAME, UMR 1137, INSERM, 75018 Paris, France; etienne.bizot@aphp.fr (E.B.); philippe.bidet@aphp.fr (P.B.); andre.birgy@aphp.fr (A.B.); patricia.mariani@aphp.fr (P.M.-K.); stephane.bonacorsi@aphp.fr (S.B.)

[2] Platform IdentyPath, Food Safety Laboratory, ANSES, Université Paris-Est, 94701, Maisons-Alfort, France; sabine.delannoy@anses.fr (S.D.); patrick.fach@anses.fr (P.F.)

[3] Centre National de Référence des *Escherichia coli*, *Shigella* et *Salmonella*, Institut Pasteur, Unités des Bactéries Pathogènes Entériques, 75015 Paris, France; francois-xavier.weill@pasteur.fr (F.-X.W.); sophie.lefevre@pasteur.fr (S.L.)

* Correspondence: aurelie.cointe@aphp.fr

Table S1. Number of ST301 sequences available in EnteroBase (on August 5, 2020) and that of those included in this study.

Main ST301 serotypes	Total no. of sequences available by serotype	Submitting country	no. of sequences submitted per country	no. of sequences included in Fig. 1	no. of sequences included in Fig. 2
O80:H2	218	France	145	4	NA
		Belgium	10	1	NA
		Germany	10	4	NA
		Poland	1	0	NA
		United Kingdom	36	6	NA
		United States of America	12	4	NA
		Spain	3	3	NA
		Switzerland	1	1	NA
O55:H9	35	France	29*	3	29
		United Kingdom	5	5	5
		Germany	1	1	1
**Other**	**48**		48	48	NA
**Total**	**301**		301	80	35

* indicates the number of strains sequenced in this study and submitted to EnteroBase. NA: not applicable

Table S2. Statistic of the 29 French O55:H9 EHEC genomes sequenced in this study.

Strain name	Strain name in EnteroBase	no. of contigs	N50	no. of bases	Coverage (X)	GenBank accession no.
**41957**	41957	389	58144	5412786	137	JAESOQ000000000
**38057**	38057	377	93977	5469344	34	JAESOP000000000
**38107**	38107	362	93736	5471004	43	JAEUBI000000000
**38108**	38108	383	71673	5469948	31	JAESOO000000000
**38110**	PMK-38110	286	110410	5327280	71	JAESON000000000
**38109**	PMK-38109	342	94386	5470482	84	JAESOM000000000
**38141**	PMK-38141	342	111326	5472775	105	JAESOL000000000
**38141delpS88**	38141delpS88	250	112303	5315838	91	JAHPZW000000000
**37541**	37541	346	98965	5488450	41	JAEUBH000000000
**44329**	44329	278	85994	5429188	158	JAESOK000000000
**46970**	46970	399	51534	5530856	289	JAESOJ000000000
**45468**	201901858	345	105264	5608734	86	JAESOI000000000
**45469**	201901859	341	111112	5607449	75	JAESOH000000000
**38868**	38868	303	75277	5433004	30	JAESOG000000000
**42850**	42850	308	67587	5183109	120	JAESOF000000000
**39224**	39224	330	74961	5438767	32	JAESOE000000000
**42605**	42605	316	65988	5414837	141	JAESOD000000000
**45509**	45509	330	65601	5412902	95	JAESOC000000000
**42592**	42592	346	64875	5488453	139	JAESOB000000000
**43000**	43000	540	29463	5359381	75	JAESOA000000000
**42262**	42262	286	74439	5411100	159	JAESNZ000000000
**43002**	43002	463	31168	5211466	110	JAESNY000000000
**44501**	201808622	359	53002	5416898	49	JAESNX000000000
**42823**	42823	336	67587	5490236	305	JAESNW000000000
**42822**	42822	380	47442	5453576	154	JAESNV000000000
**45878**	45878	343	48689	5412531	107	JAESNU000000000
**46195**	46195	432	42167	5615820	83	JAESNT000000000
**46932**	46932	271	92632	5449847	231	JAESNS000000000
**47050**	47050	303	87113	5440986	198	JAESNR000000000
